# Retraction of “Isovitexin modulates autophagy in Alzheimer’s disease via miR-107 signalling”

**DOI:** 10.1515/tnsci-2022-0262

**Published:** 2022-11-17

**Authors:** Jiang Cheng, Guowei Wang, Na Zhang, Fang Li, Lina Shi, Haining Li

**Affiliations:** Department of Neurology, General Hospital of Ningxia Medical University, Ningxia Key Laboratory of Cerebrocranial Diseases, Incubation Base of National Key Laboratory, Yinchuan, 750004, China; School of Clinical Medicine, Ningxia Medical University, Yinchuan, 750004, China

The authors’ team would like to inform that the article “Isovitexin modulates autophagy in Alzheimer’s disease via miR-107 signalling” (https://doi.org/10.1515/tnsci-2020-0109) has been retracted as per their request.

Figure 1b and Figure 7c overlap with figures of the following papers:

Chen P, Chen F, Zhou B. Antioxidative, anti-inflammatory and anti-apoptotic effects of ellagic acid in liver and brain of rats treated by d-galactose. Sci Rep. 2018 Jan;8(1):1465.

Li L, Tian J, Long MK, Chen Y, Lu J, Zhou C, et al. Protection against experimental stroke by ganglioside GM1 is associated with the inhibition of autophagy. PLoS One. 2016 Jan;11(1):e0144219.

The results are flawed and the final conclusions could not be confirmed.

